# Learning from COVID-19: The inclusion of vulnerable groups in COVID-19 responses across Europe: Jil Molenaar

**DOI:** 10.1093/eurpub/ckac129.545

**Published:** 2022-10-25

**Authors:** J Molenaar, L Van Praag

**Affiliations:** Faculty of Social Sciences, University of Antwerp, Antwerp, Belgium

## Abstract

**Background:**

More than two years into the pandemic, many European countries have begun to evaluate their COVID-19 public health responses and draw lessons for future preparedness. As the COVID-19 crisis has exacerbated intersectional social and health inequalities, it is pertinent to evaluate the extent to which COVID-19 responses have successfully responded to the diverse needs of various vulnerable groups. We present a comparative analysis of how evaluations of European COVID-19 responses have assessed efforts to adapt or tailor COVID-19 responses to vulnerable groups, focusing on public testing and tracing strategies and vaccination campaigns.

**Methods:**

We draw on data collected in the H2020 project COVINFORM. We combine insights from qualitative interviews conducted with public health policy- and decision makers in COVINFORM partner countries with a document review of available evaluations of COVID-19 responses published by both government and academic actors between March 2020 and June 2022.

**Results:**

Across countries, evaluations of COVID-19 responses show that efforts to adapt or target public health responses to specific vulnerable groups became more common as the pandemic stretched on. Differences across countries were observed in relation to which groups were considered particularly vulnerable; the types of responses considered successful; as well as the organisational/governmental level at which responses were coordinated. Analyses reveal that the heavy emphasis on medical vulnerability distracted from efforts to address broader, structural inequalities, complicating the development of tailor-made policies.

**Conclusions:**

The results inform ongoing policies that deal with the long-term consequences of the COVID-19 pandemic and aim to reduce disproportionate impacts faced by vulnerable groups. Our findings also add to a better understanding of how future preparedness structures should take into account how pandemic measures have unequal impacts.

**Key messages:**

• As the COVID-19 crisis has exacerbated intersectional social and health inequalities, it is important to learn from efforts to adapt or tailor COVID-19 responses to vulnerable groups.

• The findings demonstrate how across Europe, the combination of particular sets of country-specific COVID-19 responses, tailor-made or not, yield specific consequences for vulnerable groups.

